# Are self-reported gastrointestinal symptoms among older adults associated with increased intestinal permeability and psychological distress?

**DOI:** 10.1186/s12877-018-0767-6

**Published:** 2018-03-20

**Authors:** John-Peter Ganda Mall, Lina Östlund-Lagerström, Carl Mårten Lindqvist, Samal Algilani, Dara Rasoal, Dirk Repsilber, Robert J. Brummer, Åsa V. Keita, Ida Schoultz

**Affiliations:** 1 Nutrition Gut Brain Interactions Research Centre, Department of Medical Sciences, Faculty of Medicine and Health, Örebro University, Örebro, Sweden; 2 Nutrition and Physical Activity Research Centre, Department of Medical Sciences, Faculty of Medicine and Health, Örebro University, Örebro, Sweden; 3 Department of Clinical and Experimental Medicine, Linköping University, Linköping, Sweden

**Keywords:** Older adults, Gastrointestinal symptoms, Intestinal barrier function, Psychological distress

## Abstract

**Background:**

Despite the substantial number of older adults suffering from gastrointestinal (GI) symptoms little is known regarding the character of these complaints and whether they are associated with an altered intestinal barrier function and psychological distress. Our aim was to explore the relationship between self-reported gut health, intestinal permeability and psychological distress among older adults.

**Methods:**

Three study populations were included: 1) older adults with GI symptoms (*n* = 24), 2) a group of older adults representing the general elderly population in Sweden (*n* = 22) and 3) senior orienteering athletes as a potential model of healthy ageing (*n* = 27). Questionnaire data on gut-health, psychological distress and level of physical activity were collected. Intestinal permeability was measured by quantifying zonulin in plasma. The level of systemic and local inflammation was monitored by measuring C-reactive protein (CRP), hydrogen peroxide in plasma and calprotectin in stool samples. The relationship between biomarkers and questionnaire data in the different study populations was illustrated using a Principal Component Analysis (PCA).

**Results:**

Older adults with GI symptoms displayed significantly higher levels of both zonulin and psychological distress than both general older adults and senior orienteering athletes. The PCA analysis revealed a separation between senior orienteering athletes and older adults with GI symptoms and showed an association between GI symptoms, psychological distress and zonulin.

**Conclusions:**

Older adults with GI symptoms express increased plasma levels of zonulin, which might reflect an augmented intestinal permeability. In addition, this group suffer from higher psychological distress compared to general older adults and senior orienteering athletes. This relationship was further confirmed by a PCA plot, which illustrated an association between GI symptoms, psychological distress and intestinal permeability.

## Background

In the last decades lifespan has increased dramatically, but even though a longer lifespan is a success story in itself, it cannot be neglected that old age correlates with an increased need for health care [1]. Thus, it is important to identify areas through which a healthy ageing process can be promoted in order to increase the proportion of independent free-living older adults [2]. Gastrointestinal (GI) symptoms are common among older adults. Fifty to seventy % of older adults report symptoms of constipation [3, 4] and 4-14% experience problems with diarrhoea [5–7]. In addition, older adults suffering from diseases of the digestive system are at risk of a higher total symptom burden. [8]. Moreover, it has been established that a well functioning gut is essential in order for older adults to experience life-satisfaction and meaningfulness in everyday life [9, 10] [11]. Thus, gut health represents an important area through which health and wellbeing might be promoted. In support of this, we recently showed that senior orienteering athletes, a new potential model of healthy ageing, experience less GI symptoms than general older adults [10]. Even though age-associated GI symptoms are common in the older population, knowledge regarding the mechanisms behind these symptoms remains poor. Increased intestinal permeability is a hallmark of many GI diseases [12–14] but has not been investigated in older adults who self-report GI symptoms. The intestinal epithelium is the major interface with the external environment and while absorbing nutrients and water it simultaneously restrict the free movement of luminal material to the underlying mucosa [15, 16]. A disruption of the intestinal barrier may result in the passage of microbial antigens and toxins [17], and are associated with intestinal inflammation as well as neurological diseases [18, 19]. Furthermore, elevated levels of reactive oxygen species (ROS) (e.g. oxidative stress) have been suggested to drive intestinal inflammation [20]. Understanding the characteristics of GI symptoms in older adults and their underlying pathophysiology is important in order to facilitate diagnosis and treatment. Here, we aim to investigate how self-reported GI symptoms among older adults correlate to plasma zonulin (an indicator of increased intestinal permeability) [21, 22] and psychological distress.

## Methods

### Study participants

The study population was comprised of three groups of older adults; individuals suffering from GI symptoms (*n* = 24), senior orienteering athletes (*n* = 27) and general older adults (*n* = 22). The demographic data and recruitment process are outlined in Table 1. Comorbidities and medications are displayed in Table 2.

**Table 1:** Data for all the descriptive parameters, displayed for all three groups of older adults, presented as median (IQR)

	General older adults^a^ *n* = 22 Age: 70 (67-72)^I^	Senior orienteering athletes^b^ *n* = 27 Age: 68 (66-71)^I^	Older adults with GI symptoms^c^ *n* = 24 Age: 73 (69-75)^I^ Age at onset of GI symptoms: 59 (47-65)^II^	*p*-value^III^
- Demographic data -				
Female/Male	11/11	12/16	18/6	
Recruitment method	Advertisement in local newspaper	Orienteering clubs within the Örebro County	Advertisement in local newspaper	
Inclusion criteria	Age ≥ 65 yrs	Age ≥ 65 yrs Actively competing in orienteering	Age ≥ 65 yrs GSRS* diarrhoea/constipation^III^score ≥ 3	
Exclusion criteria	Diagnosed GI disease Prescribed inflammatory regulating drugs	Diagnosed GI disease Prescribed inflammatory regulating drugs	Diagnosed GI disease Prescribed inflammatory regulating drugs	
- Questionnaire data -				
GSRS (total score)	1.5 (1.2 – 2)	1.3 (1.1 – 1.6)	2.6 (2.2 – 3.1)	
Diarrhoea	1.3 (1 – 1.4)	1.3 (1 – 1.7)	3.3 (1.7 – 3.8)	
Constipation	1.3 (1 -2.7)	1.3 (1 – 1.7)	3.3 (2 – 4.7)	
Indigestion	1.9 (1.3 – 2.6)	1.5 (1.25 – 2)	3 (2.3 – 3.6)	
Abdominal pain	1.3 (1 – 1.8)	1	2.3 (1.7 – 3.3)	
Reflux	1 (1 – 1.5)	1	1.5 (1 – 2.5)	
HADS* (total score)	4 (1.5 – 6)	1 (0 – 3)	5 (2.5 – 10.5)	< 0.05^ab, bc^
FGAS*	4 (3-4)	4 (4-5)	4 (3-4)	
- Biomarker data -				
CRP (mg/L)	0.5 (0.2 – 1.4)	0.9 (0.2 – 1.3)	1.3 (0.7 – 2.2)	
H_2_O_2_ mmol/L	2 (1.7 – 2.2)	2 (1.7 – 2.4)	2.1 (1.8 – 2.5)	
Calprotectin (μg/g)	0 (0 – 35.9)	0 (0 – 102.5)	0 (0 – 65)	

-CRP levels < 2 are considered normal

- Calprotectin levels ≤ 50 μg/g are considered normal

- H_2_O_2_ mmol/L < 1.75 indicates *no oxidative stress*

- H_2_O_2_ mmol/L 1.75-2.35 indicates *intermediate oxidative stress*

- H_2_O_2_ mmol/L between 2.36-3.05 indicates *oxidative stress*

*-* H_2_O_2_ mmol/L 3.05 < indicates *strong oxidative stress*

* Abbreviations, *GSRS*: Gastrointestinal symptoms rating scale, *FGAS*: Frändin-Grimby Activity Scale, *HADS*: Hospital Anxiety and Depression Scale, a cut-off score of ≥8 for either subscale indicates a significant level of depression/anxiety

a = General population of older adults

b = Senior orienteering athletes

c = Older adults with GI symptoms

I: age is presented with median and interquartile range (IQR)

II: median age at onset of GI symptoms (IQR)

III: *p*-value generated from pair-wise comparisons using the Mann-Whitney U-test

**Table 2:** Distribution of comorbidities and medications used within the study groups

	Senior orienteering athletes (*n* = 27)	General older adults (*n* = 22)	Older adults with GI symptoms (*n* = 24)
Comorbidities	%	%	%
Cardiovascular diseases	14.8	18.2	50.0
Gut symptoms	11.1	22.7	100.0
Psychological & neurodegenerative morbidities	3.7	13.6	8.3
Others (respiratory tract, urinary tract, musculo-skeletal, eyes)	25.9	22.7	33.3
Medications	%	%	%
Cardiovascular agents	25.9	18.2	50.0
-Blood pressure lowering substances	18.5	18.2	37.5
-Lipid-lowering medications	- 18.5	- 0.0	- 12.5
-Anti-coagulants	- 11.1	- 0.0	- 20.8
-Others	- 3.7	- 0.0	- 12.5
Anti-inflammatory agents (NSAID, cortisone)	0	0	4.2
Gut motility regulating substances	3.7	18.2	29.2
-Anti-constipation medicine	- 0.0	- 13.6	- 12.5
-Anti-diarrheal medicine			
-Other GI regulators (probiotics, fibres)	- 7.4	- 4.5	- 4.2
-Proton pump inhibitors (PPI)	- 0.0	- 0.0	- 4.2
Antibiotics	0	4.5	16.7
-Neurology drugs	3.7	13.6	12.5
-Anti-depressants	- 3.7	- 13.6	- 0.0
-Hypnotics	- 0.0	- 0.0	- 4.2
-Dopamine-agonists	- 0.0	- 4.5	- 4.2
-Cholinesterase-inhibitors	- 0.0	- 0.0	- 4.2
Others	18.5	27.3	29.2
Polypharmacy (5 or more drugs)	3.7	0	12.5
Other GI regulators (probiotics, fibres etc.)	0	4.5	8.3

### Ethical considerations

The study obtained ethical approval by the Uppsala Regional Ethics Review Board (Dnr. 2012/309, 2013/37 and 2015/357). All procedures were carried out in accordance with the declaration of Helsinki and written informed consent was acquired from all participants.

### Questionnaires

Gastrointestinal Symptoms Rating Scale (GSRS) was used as an initial instrument to assess and define the degree of GI symptoms (Table 1). Reliability and validity of the GSRS is well documented [23]. The recruitment process was based on the GSRS score for the domains depicting diarrhoea and constipation (average score ≥ 3 of the domains for diarrhea and/or constipation) as these conditions are two of the most common GI complaints among the elderly [24, 25]. The Hospital anxiety and depression scale (HADS) is a widely used validated instrument for the evaluation of psychological distress in medical settings, as well as in older adults [26, 27]. The Frändin-Grimby Activity Scale (FGAS) was used to describe the level of physical activity [28]. Questionnaire details can be found in Table 3.

**Table 3:** Questionnaire details

	The Gastrointestinal Symptoms Rating Scale – GSRS	The Hospital Anxiety and Depression Scale - HADS	The Frändin-Grimby Activity Scale - FGAS
*Subdomains*	Diarrhoea Constipation Indigestion Abdominal pain Reflux	Anxiety Depression	Winter season Summer season
*Nr of questions*	15	14	6
*Scale*	Score 1 = No problems Score 7 = Severe problems	Score 0-3 Score 3 being the highest symptom frequency	Score 1-6
*Grading*	No problem = 1 point Mild = 1-2 points Moderate = 2-4 points Severe = 4-7 points	Normal = 0-7 points Mild = 8-10 Moderate = 11-14 Severe = 15-21	No physical activity = 1 Intense regular exercise = 6
*Total score*	Gut symptoms	Psychological distress	Physical activity

### Biological samples

#### Blood samples

After an overnight fast, the participants were instructed not to exercise in the morning, before collection of the blood samples. Plasma from senior orienteering athletes was separated using the Ficoll-paque PLUS (GE Healthcare Life Sciences, UK). For older adults suffering from GI problems and general older adults, plasma was separated using standard operating procedures used in the daily routine at the Örebro University Hospital. Blood samples were centrifuged at 2200 g for 10 min and plasma was collected in Eppendorf tubes. Blood samples from 10 individuals were collected using both separation procedures and subsequently analysed to evaluate the difference between the two techniques; in the following analyses the difference was controlled for.

***Systemic inflammation.*** **C-reactive protein (CRP) is an acute-phase plasma protein and a marker of inflammation.** CRP levels were assessed by the high-sensitivity immunoturbidimetric assay, CardioPhase™, and analysed on the ADVIA 1800 chemistry system (SIEMENS Healthcare Diagostics Inc., NY, USA). A normal CRP was defined as ≤2 mg/L***.***

***Oxidative stress.*** **Oxidative stress was estimated by the FORT (Free Oxygen Radicals Test)** colorimetric assay (Callegari, Parma, Italy), as previously described [29].

***Intestinal permeability.*** To examine zonulin levels in plasma, as an indirect indicator of small intestinal permeability [22], a zonulin ELISA kit was used following manufacturer’s instructions (Cusabio, Baltimore, USA). In brief, plasma was diluted 1:2, and added to pre-coated plates in duplicate, together with controls and standards. Primary biotinylated antibody, streptavidin-horseradish peroxidase, tetramethylbenzidine enzyme substrate, and 2 M HCL were subsequently added. Absorbance was measured at 450 nm in VERSAmax Tunable Microplate Reader (Molecular Devices, San Diego, CA, USA) using Softmax pro 5 (Molecular Devices). The software generated a standard curve based on the standards, from which the concentrations of the samples were calculated. The detection range of the kit was 0.625 ng/ml – 40 ng/ml, with the lower limit of detection at 0.156 ng/ml according to the manufacturers information. In addition, the quantification and intra-assay precision (CV%) was < 8%.

#### Stool samples

Material for faecal sample collection was provided together with comprehensive instruction one-week prior to collection. The sample was collected at the participant’s home, instantly frozen at -20 °C and transported to the laboratory within one week. Samples were stored at -80 °C until analysis.

***Intestinal inflammation.*** Faecal calprotectin was measured to assess intestinal inflammation using the ELISA assay CALPRO® (CALPRO AS, Lysaker, Norway). Approximately 100 mg of faeces were extracted in 5 mL of extraction buffer and analysed using a pre-coated 96-well plate provided by the manufacturer. The enzyme reaction was detected at 405 nm. The normal range for faecal calprotectin is defined as < 50 μg/g.

### Statistical methods & data analysis

All data are presented with median and IQR. Shapiro-Wilk test was used to assess normality. The non-parametric Kruskal-Wallis test was used to identify statistical differences between the three study groups, followed by the non-parametric Mann-Whitney U-test for pair-wise comparisons. The Bonferroni-Holm method was used to correct for multiplicity. All statistical analyses were performed using GraphPad Prism version 6 for Mac (GraphPad Software, San Diego California, USA). For visualization of the data biplots of Principal Component Analysis (PCA) were created using R v3.3.0 [30] with functions prcomp and ggbiplot. By convention all values below detection levels were replaced with 0 to facilitate the analysis.

## Results

### Intestinal permeability

**Fig. 1:**
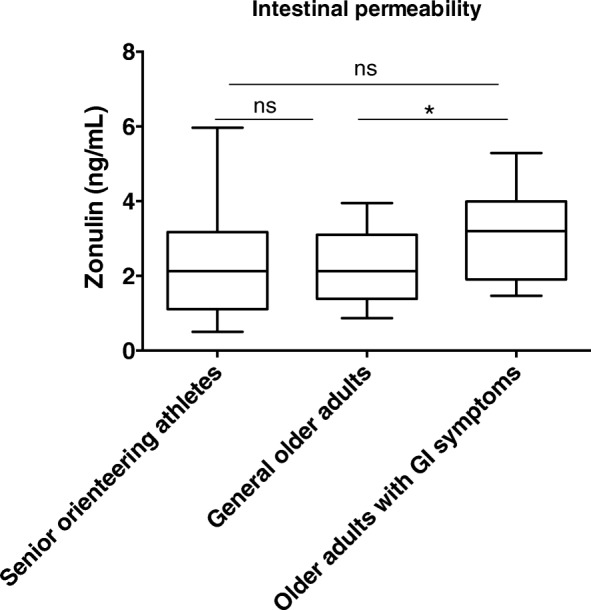
Intestinal permeability as reflected by plasma zonulin levels in the different groups of older adults. The group of older adults with GI symptoms (*n* = 22) had significantly higher plasma zonulin levels than and general older adults (*n* = 21) but not senior orienteering athletes (*n* = 27). No significant difference was observed between senior orienteering athletes and general older adults. **p < 0.01, *p < 0.05

Assessment of zonulin, an indirect marker of intestinal permeability, showed significantly increased levels among older adults suffering from GI symptoms, compared to general older adults (*p* < 0.05) but not to senior orienteering athletes (Fig. 1).

### Psychological distress

**Fig. 2:**
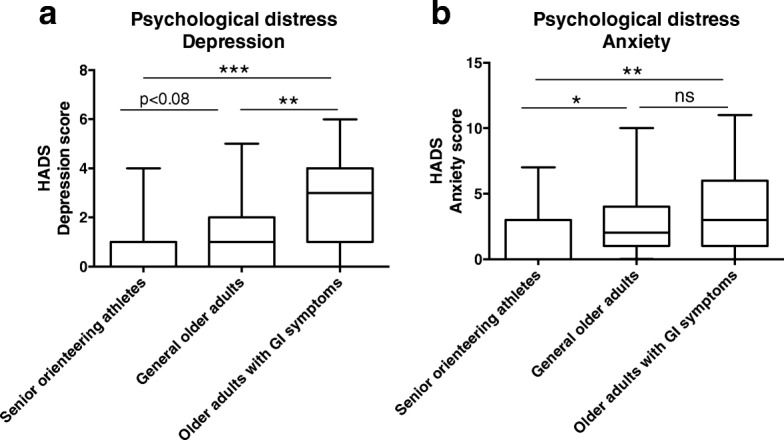
Depression and anxiety assessed by HADS in the three different populations of older adults. **a**) The group experiencing GI symptoms (*n* = 24) revealed significantly higher depression scores on the HADS scale than the other two groups. A trend towards a significant difference was observed between the senior orienteering athletes (n = 27) and general older adults (n = 22). **b**) Anxiety score was significantly higher in older adults with GI symptoms compared to the senior orienteering athletes. A significantly lower score was seen in senior orienteering athletes when compared to general older adults, with the latter showing no difference compared to group experiencing GI symptoms. *p < 0.05; **p < 0.01; ***p < 0.001, ns = non-significant

Assessment of overall psychological distress (total HADS score) showed increased levels for both older adults with GI symptoms (p < 0.05) and general older adults (*p* < 0.05) when compared to senior orienteering athletes (Table 1). The level of probable depression was found to be significantly higher among older adults with GI symptoms compared to both general older adults (*p* < 0.01) and senior orienteering athletes (*p* < 0.001) (Fig. 2a). Experience of anxiety showed a different pattern and was found to be significantly higher in both older adults with and without GI symptoms (p < 0.05) compared to senior orienteering athletes (Fig. 2b).

### Biomarkers of inflammation and oxidative stress

Biomarkers of inflammation and oxidative stress were assessed to monitor the inflammatory status of the study participants. For all three populations the CRP levels were found to be within the normal range, < 2 mg/L. Similarly, values of oxidative stress and faecal calprotectin were found to be within the normal range. These data are presented as descriptive values in Table 1.

### Comorbidities and medication

Comorbidities and medical use were reported in the case report forms and are presented in Table 2. The usage of cardiovascular drugs (blood pressure lowering drugs, anti-coagulants etc) was most prominent in all groups. Fifty percent of older adults with GI symptoms reported cardiovascular disease and intake of cardiovascular drugs. Hypertension was identified as the most common condition. Older adults with GI symptoms were also found to consume gut motility regulating substances (29.2%) to a larger extent compared to the two other study groups. Use of antibiotics was also found to be higher in the study group suffering from GI symptoms. Polypharmacy (defined as intake of five or more medications simultaneously) was found to be more prominent in older adults suffering from GI symptoms (12.5%) compared to both senior orienteering athletes (3.7%) and general older adults (0%). The levels of zonulin and psychological distress was further found not be influenced by medication and comorbidities when confounding factors were controlled for.

### Illustration of the data using principal component analysis

**Fig. 3:**
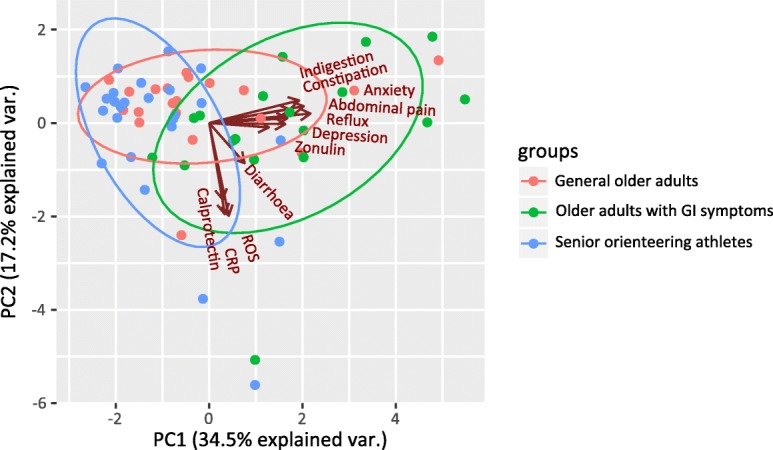
Principal component analysis displaying the relationship between all investigated biomarkers, HADS domains and GSRS domains in the 3 study populations. Both zonulin and HADS appear associated with the GSRS domains unlike ROS, CRP and calprotectin. The ellipsoid markings cover 95% of the total populations

The PCA revealed a separation between the 3 different study populations, based on GSRS scores and biomarkers (Fig. 3). The highest degree of separation was observed between the senior orienteering athletes and older adults with GI symptoms. The general population of older adults were found to clearly overlap the other two populations. This separation is expected and can be explained by the higher GSRS score on the domains diarrhoea and/or constipation among older adults with GI symptoms. An association could be observed between C-reactive protein, ROS and calprotectin. In addition, GI symptoms appear to be associated with zonulin and psychological distress.

## Discussion

To our knowledge this is the first study that investigated the characteristics of self-reported GI symptoms. This is important to facilitate treatment and diagnosis for older adults experiencing moderate GI symptoms. No treatment regimens solely designed for age-associated GI symptoms exist today, and knowledge regarding gut and intestinal barrier function in older adults is poor. As a first step to identify the mechanisms behind age-associated GI symptoms we investigated the relationship between self-reported GI symptoms and intestinal permeability. Furthermore, the impact of GI symptoms on wellbeing was assessed, by investigating the level of psychological distress in relation to experience of gut problems.

As outlined in the results section, we identified that depression-like characteristics were more prominent among older adults suffering from GI symptoms and less common among senior orienteering athletes. The low level of psychological distress among senior orienteering athletes is in line with our previous results [10]. Senior orienteering athletes were also found to suffer from less GI symptoms. Experience of anxiety did not differ between older adults with and without GI symptoms. An increased prevalence of anxiety disorders among older adults has been reported previously [31–33]. However, the levels of psychological distress estimated by HADS were below the cut-off value (≥ 8) for severe anxiety and depression. Hence, indicating that none of the study participants suffered from severe psychological disease. It should also be taken into consideration that the results presented here are based on self-reported data, which relied on the respondents’ honesty, accuracy, and interpretation of the question asked.

GI symptoms were found to be associated with elevated levels of plasma zonulin, which indicate that older adults suffering from GI symptoms have increased small intestinal permeability. Moreover, the PCA analysis showed that GI symptoms were found to be associated with psychological distress and zonulin among older adults with GI symptoms. Females (*n* = 18) were further overrepresented among elderly suffering from GI symptoms (*n* = 24). This could reflect the normal distribution of GI symptoms in the population as previous studies report an increased frequency of GI symptoms, such as constipation, among women [34–36]. Stratification for gender did not reveal a significant difference between men and women in regard to GI symptoms, zonulin levels and depression-like characteristics. Anxiety-like characteristics were, on the contrary, found to be significantly higher among elderly men with GI symptoms (*n* = 6). However, this finding could be due to the small study population and further studies using larger sample sets will need to be performed in order to thoroughly elucidate the difference between men and women in relation to psychological distress, GI symptoms and intestinal barrier function.

Increased intestinal permeability is a hallmark in the pathophysiology of chronic inflammatory gastrointestinal diseases, such as Crohn’s disease [13]. An altered intestinal permeability has previously been associated with psychiatric disorders such as depression and anxiety [37]. Thus, our results support the notion that intestinal permeability might be an important target for new treatment regimes for age-associated GI symptoms that might have a positive impact on mental wellbeing. Moreover, elderly individuals are known to have a low fibre intake [38] that in addition to a disturbed intestinal motility could alter the gut microbiota and result in a diminished diversity that could have a negative impact on the intestinal barrier function [37]. Recently, we showed that a dietary fibre from yeast was able to attenuate stress-induced hyperpermeability ex vivo across small intestinal tissue from Crohn’s disease patients mounted in the Ussing Chamber [39]. Thus, dietary fibres could be a potential therapeutic able to strengthen the intestinal barrier in elderly individuals, however, this needs to be thoroughly investigated in pre-clinical and clinical settings.

Zonulin is the only physiological mediator known to reversibly regulate intestinal permeability by modulating intercellular tight junctions [21, 40]. Circulating zonulin in serum/plasma is considered a useful marker of small intestinal permeability [21, 41] and has been validated using lactulose/mannitol tests [22]. However, zonulin as a marker of small intestinal permeability has been, and is, under debate. Levels of zonulin have been found to fluctuate over time making interpretation of the results difficult [42]. Moreover, a recent study suggests that circulating zonulin might not only be derived from the gastrointestinal tract but may be associated with obesity and hyperlipidaemia [43, 44]. However, the body mass index (BMI) of the general older adults (25.6 ± 4.3 Std) and older adults with GI symptoms (26.7 ± 5.0 Std) included in the present study was normally distributed and no significant differences were observed between the two groups. In addition, stratification of the data revealed no association between cardiovascular disease, including hypertension, and increased zonulin levels. Unfortunately, the BMI was not available for the senior orienteering athletes in the present study. However, the BMI of eleven newly recruited senior orienteering athletes enrolled in an additional study was found to be normally distributed with a mean value of 23.8 ± 3.2 Std. This is in accordance with a recent study showing a lower BMI value of senior athletes compared to general older adults [45]. Hence, these findings indicate that the increased zonulin levels in the present study were not a result of overweight/obesity or cardiovascular disease.

Nevertheless, the findings presented here needs to be confirmed in future studies using more advanced techniques, such as the Ussing Chamber methodology. This will allow for a thorough assessment ex vivo of the intestinal barrier function in elderly using mucosal biopsies [12, 39, 46] and hence add important information to the results presented here.

Moreover, it is important to point out that older adults with GI symptoms were found to suffer from more comorbidities and did also use more medications. Fifty percent were identified to suffer from cardiovascular disease, were hypertension was found to be the most common condition. A low dose (75 mg) of acetylsalicylic acid (ASA) is commonly prescribed to treat hypertension [47, 48]. ASA is known to affect the intestinal barrier negatively and induce an increased permeability [49]. In addition, beta-adrenoceptor blocking agents (beta-blockers, mainly used to treat angina pectoris) have been found to decrease bacterial translocation [50]. However, only three participants reported use of ASA and two used beta-blockers. Moreover, the median plasma zonulin value in the participants taking medications did not differ from the median of the whole study group. Antibiotic use was also higher among the participants suffering from GI symptoms (16.7%). Antibiotic use is known to influence the gut microbiota negatively and can cause antibiotic associated diarrhoea [51, 52]. Interestingly, three out of four participants suffered from diarrhoea. However, none of these participants displayed zonulin levels above the median for older adults suffering from GI symptoms. Moreover, it is important to point out that assessment of the gut microbiota was not performed in the present study. Thus, we cannot exclude that the increased intestinal permeability is a consequence of an altered gut flora with an increased number of pathobionts. Nevertheless, assessment of confounding effect showed that medication and comorbidities did not influence intestinal permeability or psychological distress.

In addition, assessment of biomarkers showed no change in inflammatory status in regard to GI symptoms. Thus, confirming that none of the study participants suffered from severe inflammation and infection. It should, however, be noted that in order to thoroughly assess low-grade inflammation pro-inflammatory cytokines, such as IL-6, TNF-α and IL-1β, should be investigated. Moreover, absence of intestinal inflammation was confirmed by the low calprotectin levels, which fell within the normal range. Thus, indicating that the increased intestinal permeability was not dependent on intestinal inflammation. Hence, our data suggest that moderate GI symptoms area associated with an altered intestinal barrier function and psychological distress. However, in future studies it will be important to perform thorough analysis of the gut microbiota and assess intestinal barrier function using more advanced technology.

## Conclusions

Here we demonstrate that self-reported GI symptoms among older adults are associated with an altered intestinal barrier function as assessed by plasma zonulin levels. Furthermore, both GI symptoms and zonulin levels are associated with increased psychological distress. These findings suggest that even moderate levels of GI symptoms can be reflected in a disturbed intestinal barrier function and increased psychological distress.

## Abbreviations

ASA: Acetylsalicylic acid
CRP: C-reactive protein
FGAS: Frändin-Grimby activity scale
FORT: Free oxygen radicals test
GI: Gastrointestinal
GSRS: Gastrointestinal symptoms rating scale
HADS: Hospital anxiety and depression scale
PCA: Principal component analysis
ROS: Reactive oxygen species
